# Monophosphoryl lipid a attenuates radiation injury through TLR4 activation

**DOI:** 10.18632/oncotarget.20907

**Published:** 2017-09-15

**Authors:** Jiaming Guo, Yuanyuan Chen, Xiao Lei, Yang Xu, Zhe Liu, Jianming Cai, Fu Gao, Yanyong Yang

**Affiliations:** ^1^ Department of Radiation Medicine, Faculty of Naval Medicine, Second Military Medical University, Shanghai, 200433, P.R. China

**Keywords:** monophosphoryl lipid a (MPLA), radioprotection, toll like receptor 4 (TLR4), TRIF, MyD88

## Abstract

Ionizing radiation causes severe damage to human body, and normal tissue toxicity in cancer radiotherapy also limits its further application. It is urgently required to develop safe and effective radioprotector. Our previous study has shown that toll like receptor 4 (TLR4) was dispensable for basal radiation resistance. However, severe toxicity of its traditional agonist lipopolysaccharide limits the clinical application. In present study, we demonstrated that monophosphoryl lipid A (MPLA), a potent TLR4 agonist with low toxicity, effectively attenuated radiation injury on *in vitro* and *in vivo*. MPLA increased cell survival and inhibited cell apoptosis after irradiation, and cell cycle arrest was also inhibited. Radiosensitive tissues including spleen, intestine, bone marrow and testis were protected from radiation damages in a TLR4 dependent manner. We also found that myeloid differentiation factor 88 (MyD88) accounted more than Toll/IL-1R domain-containing adaptor inducing IFN-β (TRIF) for the radioprotective effects of MPLA. In conclusion, our finding suggests TLR4 agonist MPLA as a safe and effective radioprotector for clinical application.

## INTRODUCTION

Acute high dose radiation exposure could result from multiple potential disaster scenarios, such as nuclear reactor meltdown, dirty bomb, or nuclear bomb explosion *etc.* [1]. Side effects on normal tissues caused by cancer radiotherapy are also barriers to further application of higher doses of radiation. It is critical to prevent further injury and protect the rescuers by using radioprotectors before irradiation [2, 3]. The current radioprotectors mainly fall into two categories: immunomodulators /cytokines/growth factors; antioxidants/free radical scavengers [4]. But the efficacy and toxicity of these agents limits further applications in radiation protection. It is urgently required to develop safe and effective radioprotectors to protect against radiation injury.

Recently, Toll like receptors (TLRs) had been shown to exhibit radioprotective effects in mice as well as monkeys [5-7]. Our department found that mice deficient in TLR4 were more susceptible to radiation-induced mortality and morbidity [8]. And TLR4 *in vivo* ligand, lipopolysaccharide (LPS), significantly increased animal survival in response to lethal dose irradiation [8]. However, the toxicity of LPS in multiple tissues limits its application. Alternative TLR4 agonist with low toxicity is required.

Monophosphoryl lipid A (MPLA), a potent TLR4 agonist, is currently used as a vaccine adjuvant in clinics [9-11]. MPLA is produced by hydrolysis of native diphosphoryl lipid A, which retains the component of LPS recognized by TLR4. This structural changes of MPLA decrease systemic toxicity by >99% compared with native lipid A [12], and MPLA is 10,000 times less toxic than LPS [13]. And over 300,000 human tests in vaccine have proved the safety of MPLA [14]. In present study, we aimed to investigate the protective effects of MPLA against ionizing radiation. And we demonstrated that MPLA effectively protected cultured cells and mice in a TLR4-MyD88 dependent manner.

## RESULTS

### MPLA protected cells from apoptosis and DNA damage induced by IR

**Figure 1:**
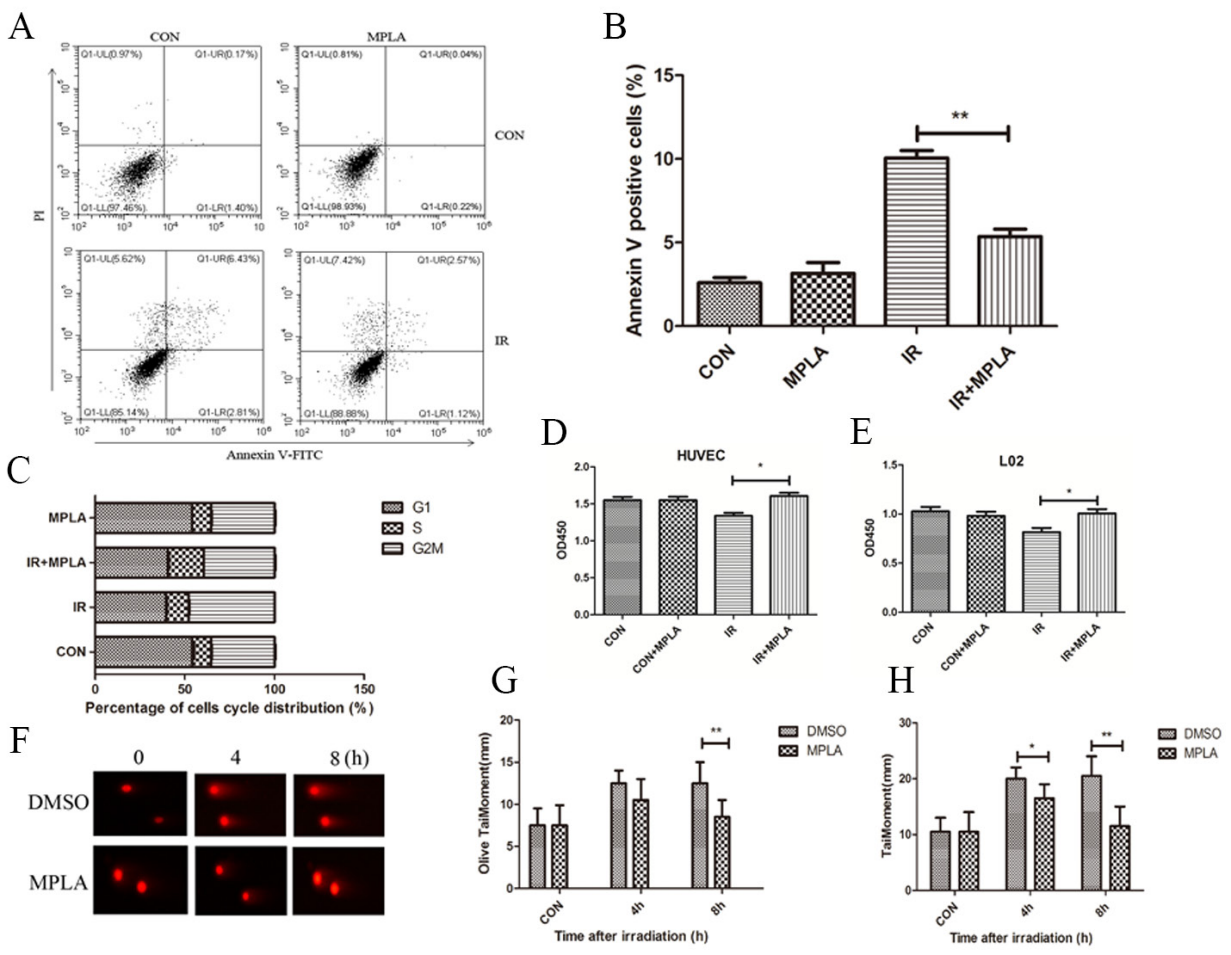
MPLA protected cells against cell death and alleviated DNA damage induced by IR RAW264.7 cells were treated with MPLA (1ug/mL) before 6Gy irradiation, at 24h after which cell apoptosis was determined with flow cytometry **(A)** and percentages of Annexin V positive cells were quantified **(B)**. Cell cycle distribution was also analyzed at 12h after irradiation **(C)**. Cell survival of HUVEC **(D)** and L02 cells **(E)** were analyzed at 48h post-irradiation by using a CCK-8 method. Comet assay was used to measure DNA damage of irradiated cells at different time points **(F)**. Olive tail moment **(G)** and tail moment **(H)** were quantified. ^*^P<0.05, ^**^P<0.01 Vs IR groups. (n=6)

In HUVEC and L02 cells, we found that IR significantly induced apoptosis at 24h and G2/M cell cycle arrest at 12h (Figure 1A, 1B, 1C). In MPLA pretreated group, apoptosis and cell cycle arrest was significantly inhibited (Figure 1A, 1B, 1C), and cell survival significantly increased (Figure 1D, 1E). These data indicates that MPLA exhibit protective effects in cultured cells against IR. By using a comet assay, we found that MPLA alleviated DNA damage induced by IR, and significantly reduced olive tail moment and tail moment (mm) (Figure 1F, 1G, 1H).

### MPLA activates NF-kB and regulated MAPK signaling pathway

**Figure 2:**
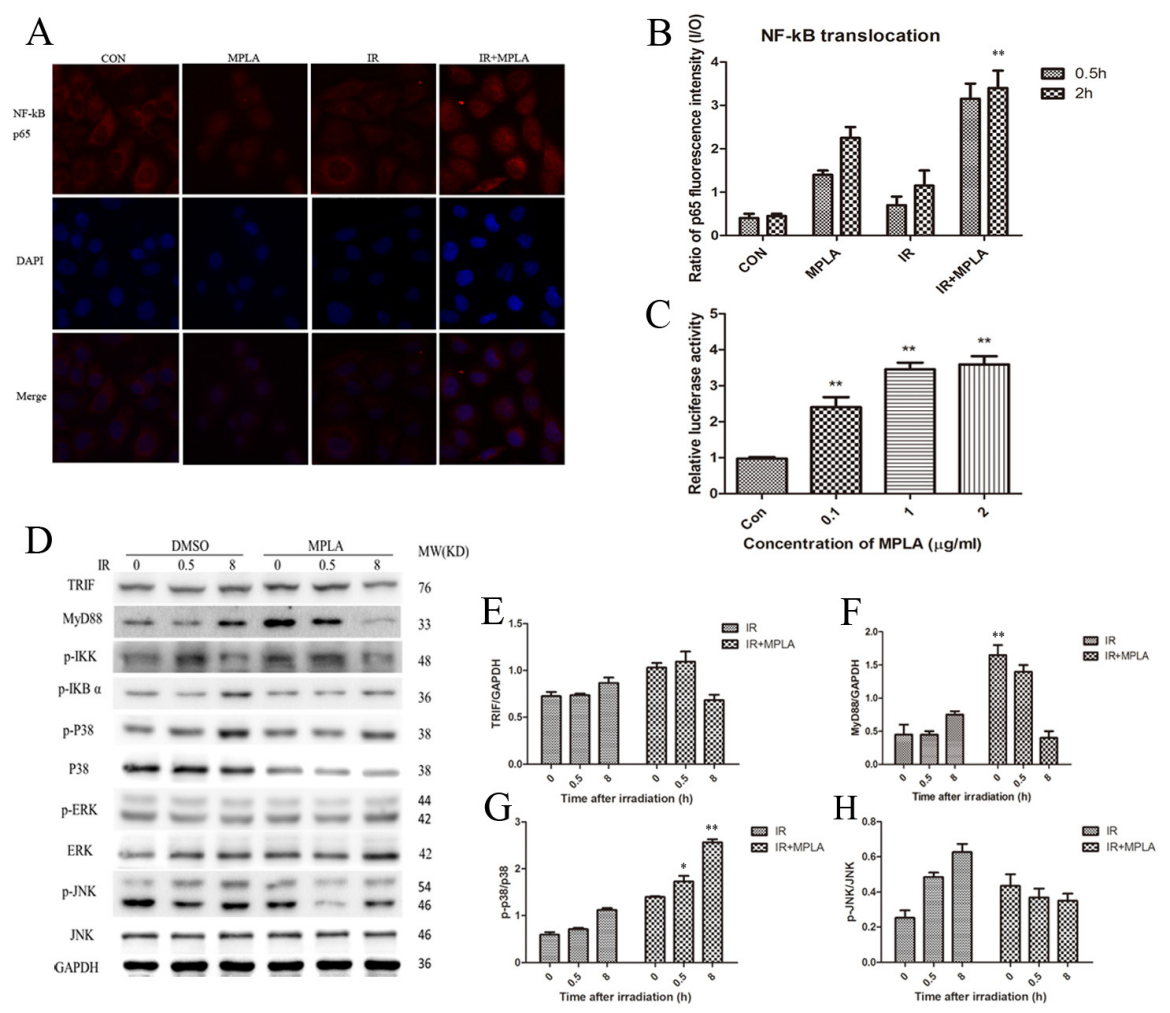
MPLA induced translocation of NF-kB p65 and activated MAPK signaling pathway At 0.5h and 2h after 8Gy irradiation, HUVEC cells were stained with NF-kB p65 antibody **(A)** and p65 fluorescence inside and outside the nuclear were quantified and the ratio was calculated **(B)**. NF-kB luciferase activity was measured in 293T cells after different centration of MPLA treatment **(C)**. Western blot assay of TRIF, MyD88, and MAPK signaling pathway were examined at 0, 0.5 and 8h after irradiation **(D)**. Raw density of TRIF **(E)**, MyD88 **(F)**, p-p38 **(G)** and pJNK **(H)**, compared to internal control, was analyzed. **P<0.01 Vs IR groups. (n=8)

NF-kB p65 was assumed accounting for radio-protection of TLRs. Our data showed that MPLA pretreatment induced significant translocation of NF-kB p65 into the nucleus (Figure 2A, 2B). MPLA treatment also enhanced the activity of NF-kB (Figure 2C). TRIF and MyD88 are two main adaptors mediating the signaling from TLR4, and thus activating MAPK signaling pathway. We found that MPLA treatment significantly increased the level of MyD88 (Figure 2D, 2F). The phosphorylation of MAPK pathway was also examined, in which we found that MPLA induced phosphorylation of p38 and IKK-β, but suppressed the phosphorylation of JNK (Figure 2D, 2G, 2H).

### MPLA protected bone marrow and increased nucleated cells against irradiation

**Figure 3:**
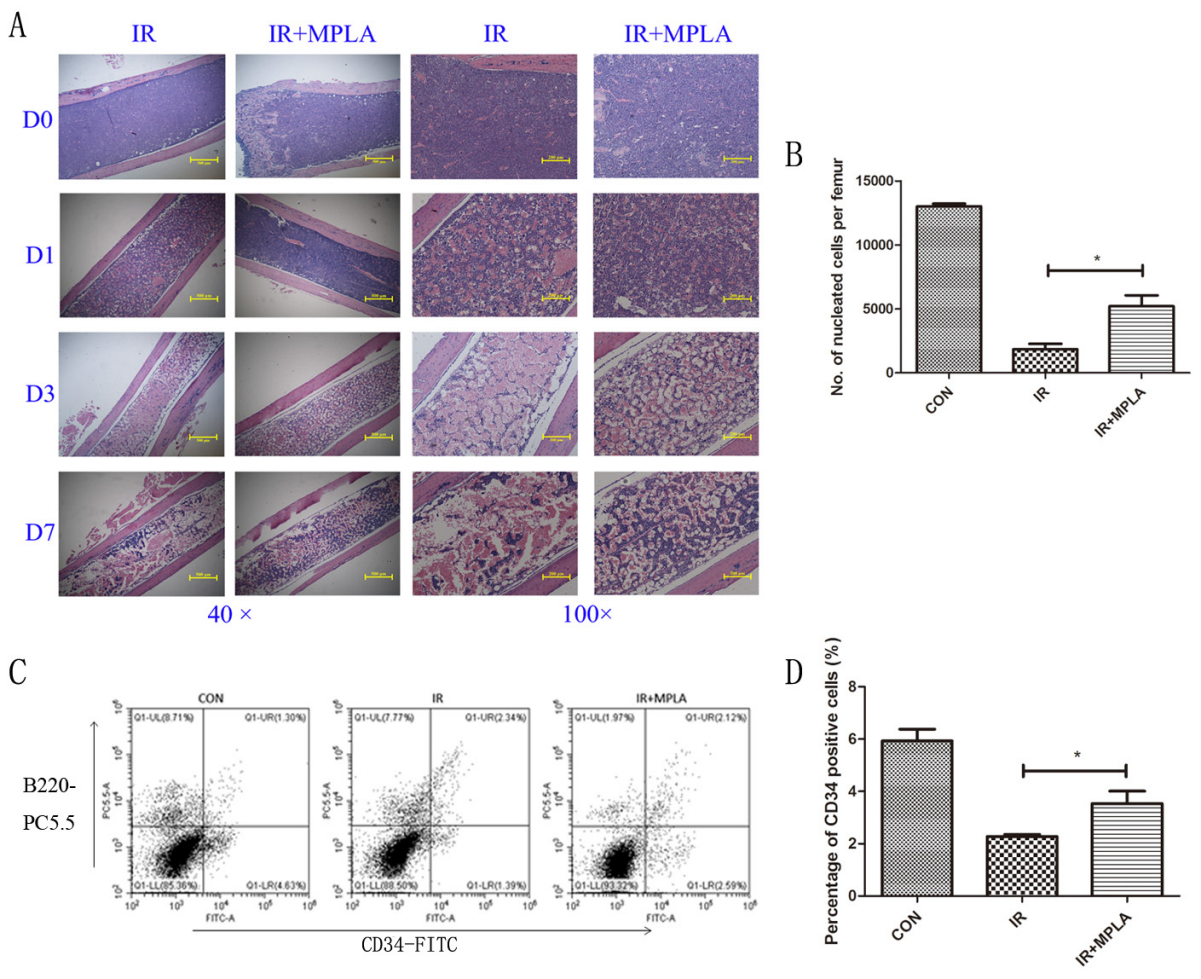
MPLA alleviated destructions of bone marrow and increased bone marrow nucleated cells following IR On Day 0, Day 1, Day 3, and Day 7 post 7Gy-irradiation, femurs were isolated and subjected to HE staining **(A)**. On the third day after irradiation, bone marrow nucleated cells **(B)** and CD34^+^ HSC was analyzed with flow cytometry using CD34-FITC and B220-PC5.5 antibodies **(C)**. The percentages of CD34-FITC^+^ cells were quantified **(D)**. *P<0.05, **P<0.01 Vs IR groups. (n=8).

To examine the radioprotective effects of MPLA *in vivo*, we checked the changes of radiosensitive tissues including bone marrow, spleen, intestine and testis. In bone marrow, we observed progressive destruction of bone marrow in the single irradiated group, and the decrease of nucleated cells was significantly inhibited by MPLA treatment (Figure 3A, 3B). We also found that MPLA treatment increased the number of CD34^+^ HSC in bone marrow (Figure 3C, 3D).

### MPLA alleviated radiation damages on spleen and immune imbalance

**Figure 4:**
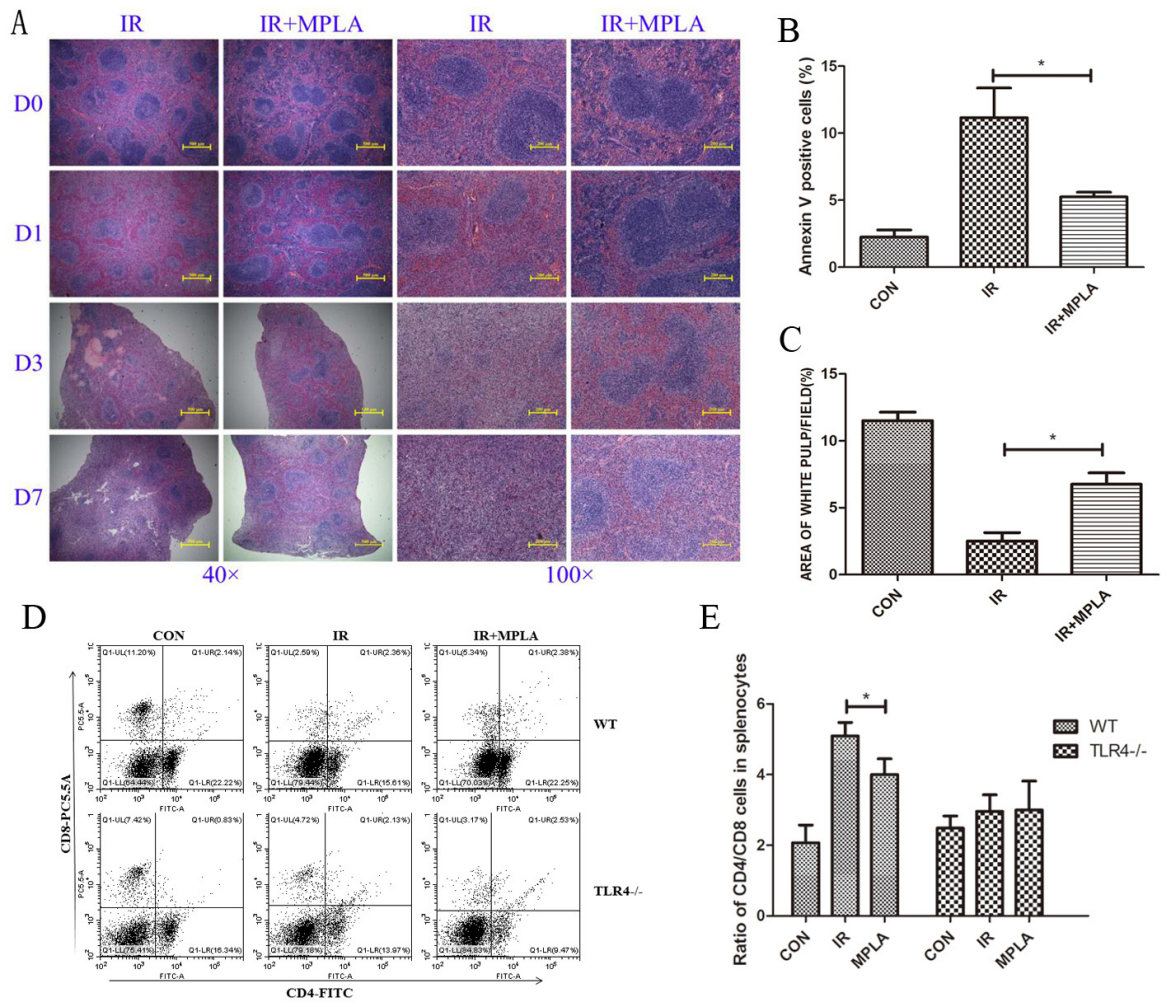
MPLA alleviated radiation damages on spleen and CD4/CD8 immune imbalance On Day 0, Day 1, Day 3, and Day 7 post 7Gy-irradiation, spleen from different groups were isolated and subjected to HE staining **(A)**. Splenocytes in single cells suspension was prepared and analyzed for cell apoptosis in on Day 3 post-irradiation **(B)**. The area of white pulps in each group was calculated with Image J software **(C)**. Percentages of CD4^+^ and CD8^+^ cells in splenocytes were analyzed **(D)** and ratio of CD4+ and CD8+ cells in wild type and TLR4 knockout mice **(E)**. *P<0.05, **P<0.01 Vs IR groups. (n=5)

After irradiation, size of white pulps reduced obviously compared to the control groups. But in MPLA-treated groups, area of white pulps were significantly retained (Figure 4A, 4C). MPLA also significantly inhibited radiation-induced apoptosis in splenocytes (Figure 4B). We examined the ratio of CD4^+^ and CD8^+^ cells in spleen, and found MPLA significantly reversed the imbalance of CD4^+^/CD8^+^ T cells, which was severely disrupted by IR (Figure 4D, 4E).

### MPLA suppressed the inflammation cytokines induced by irradiation

**Figure 5:**
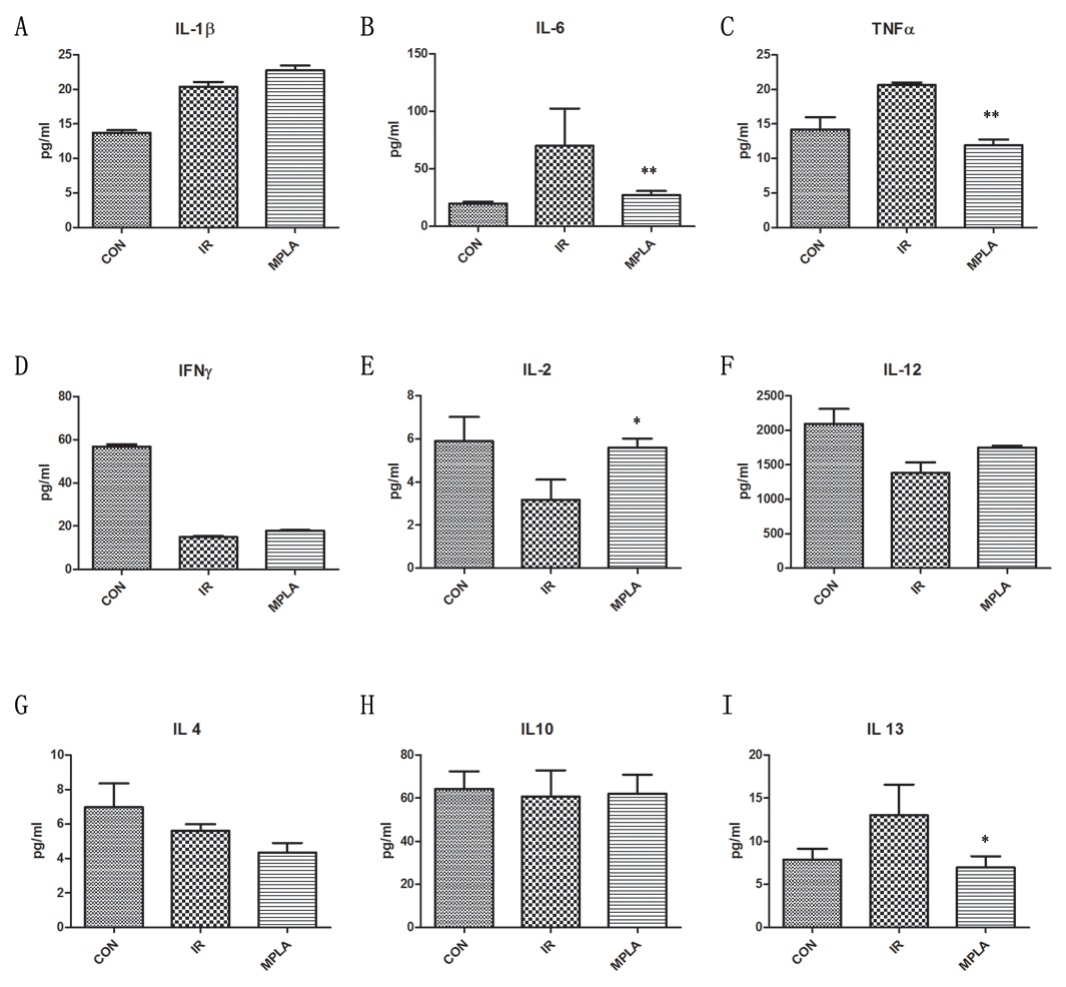
MPLA suppressed the inflammation cytokines induced by irradiation On the third day after 7Gy irradiation, blood serum of mice from different groups was isolated. Concentrations of cytokines in blood serum were measured by Elisa assay on the third day after irradiation **(A-I)**. *P<0.05, **P<0.01 Vs IR groups. (n=6)

IR often causes burst of inflammatory cytokines and imbalance of Th1/Th2 immunity. We found that MPLA suppressed the level of TNF-α (Figure 5C) and IL-6 (Figure 5B), which were upregulated by IR, with no effect on IL-1β (Figure 5A). IR caused decrease of Th1-related cytokines and elevated Th2-related cytokines. We found that MPLA significantly improved the level of IL-2 (Figure 5E) and IL-12 (Figure 5F), while downregulated the levels of IL-13 (Figure 5I). These data suggests that MPLA exerts anti-inflammatory and immunomodulatory effects in response to IR.

### MPLA protected testis and intestinal damages induced by irradiation and increased animal survival

**Figure 6:**
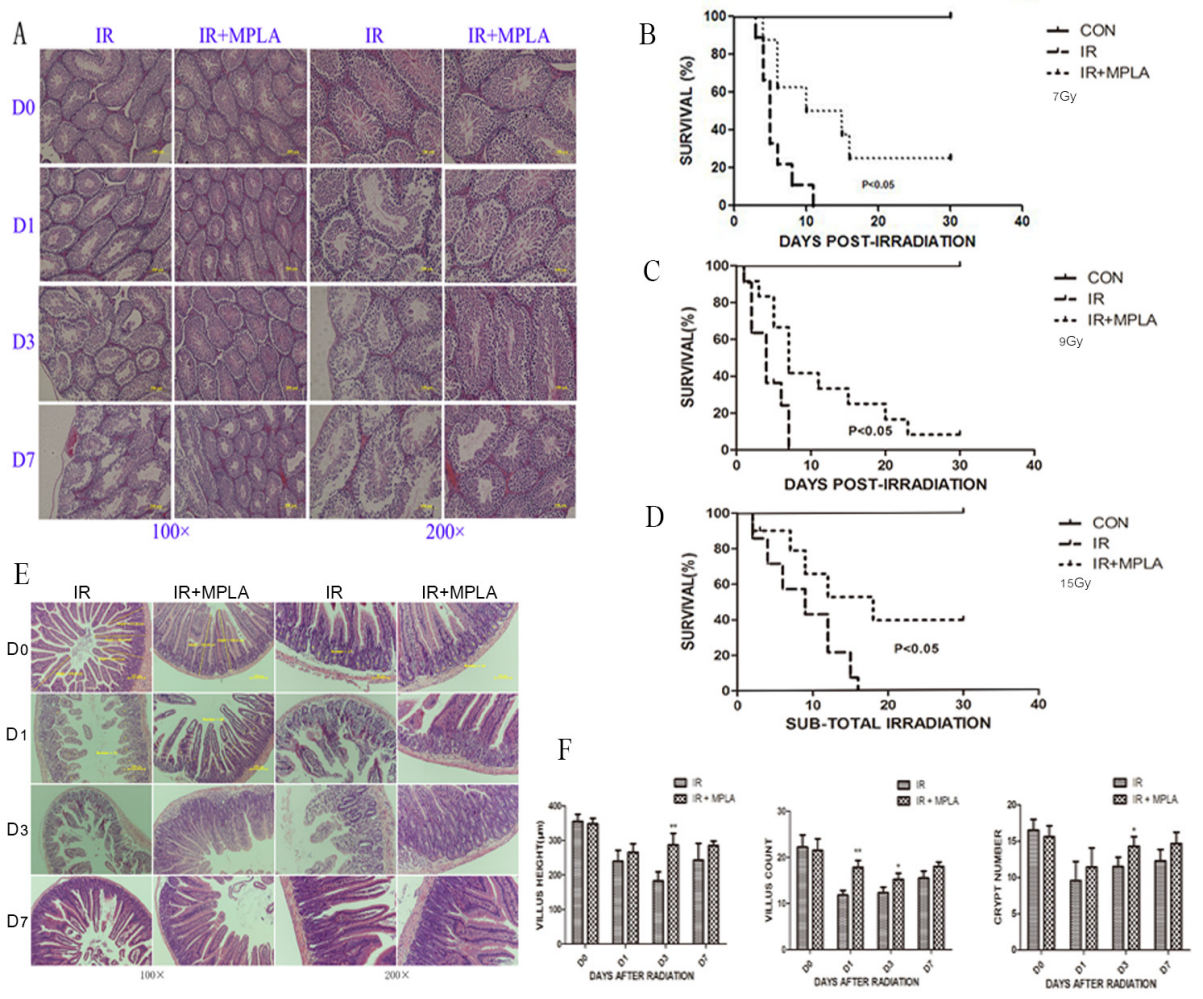
MPLA protected testis and intestinal damage and increased animal survival in response to IR On different day after 7Gy total-body irradiation, testis **(A)** and small intestine **(E)** were isolated and stained with a HE staining method. Villus height, villus number and the number of crypt in intestine were quantified and analyzed **(F)**. Survival of mice with and without MPLA pretreatment was monitored up to 30 days post 7Gy **(B)** or 9Gy **(C)** total body irradiation. We tested survival of mice exposed to 15Gy sub-total irradiation **(D)**. *P<0.05, Vs IR groups. (n=10)

Male reproductive system is very sensitive to IR, and we found that irradiation resulted in severe structural damage and progressive germ cell loss (Figure 6A). In MPLA pretreated group, the structural damage of testis and intestine was protected, especially the progressive damages in latter time (Figure 6A, 6E, 6F). MPLA treatment inhibited villi and crypt loss of intestine caused by IR (Figure 6B). We also proved that MPLA pretreatment increased survival in mice after 7Gy and 9Gy total-body irradiation (Figure 6B, 6C). MPLA also protected mice from 15Gy sub-total irradiation (Figure 6D).

### Radioprotective effects of MPLA were abrogated in TLR4-/- mice but not in TRIF mutant mice

**Figure 7:**
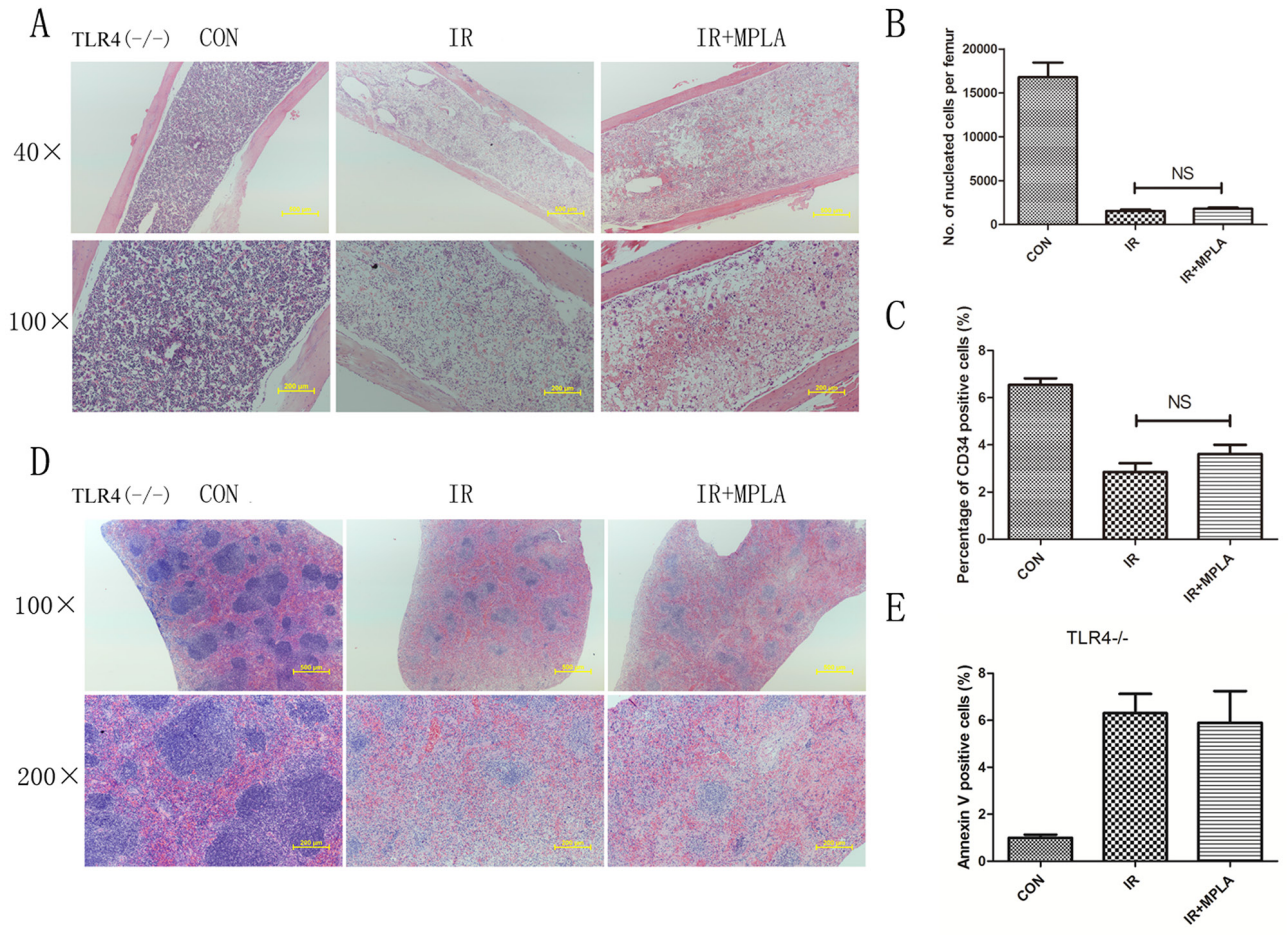
Radioprotective effects of MPLA were abrogated in TLR4-/- mice TLR4 knockout mice were exposed to 7Gy total-body irradiation, after then femur and spleen were isolated on the third day. Femur **(A)** and spleen **(D)** in TLR4-/- mice were subjected to HE staining of sections from on Day 3 post-irradiation. Bone marrow nucleated cells in TLR4-/- mice were also analyzed from different groups **(B)**. Percentages of CD34+ HSC in bone marrow cells from different groups were counted **(C)**. Flow cytometry assay was also used to measure splenocytes apoptosis from TLR4-/- mice **(E)**. NS, non significant Vs IR groups. (n=5)

**Figure 8:**
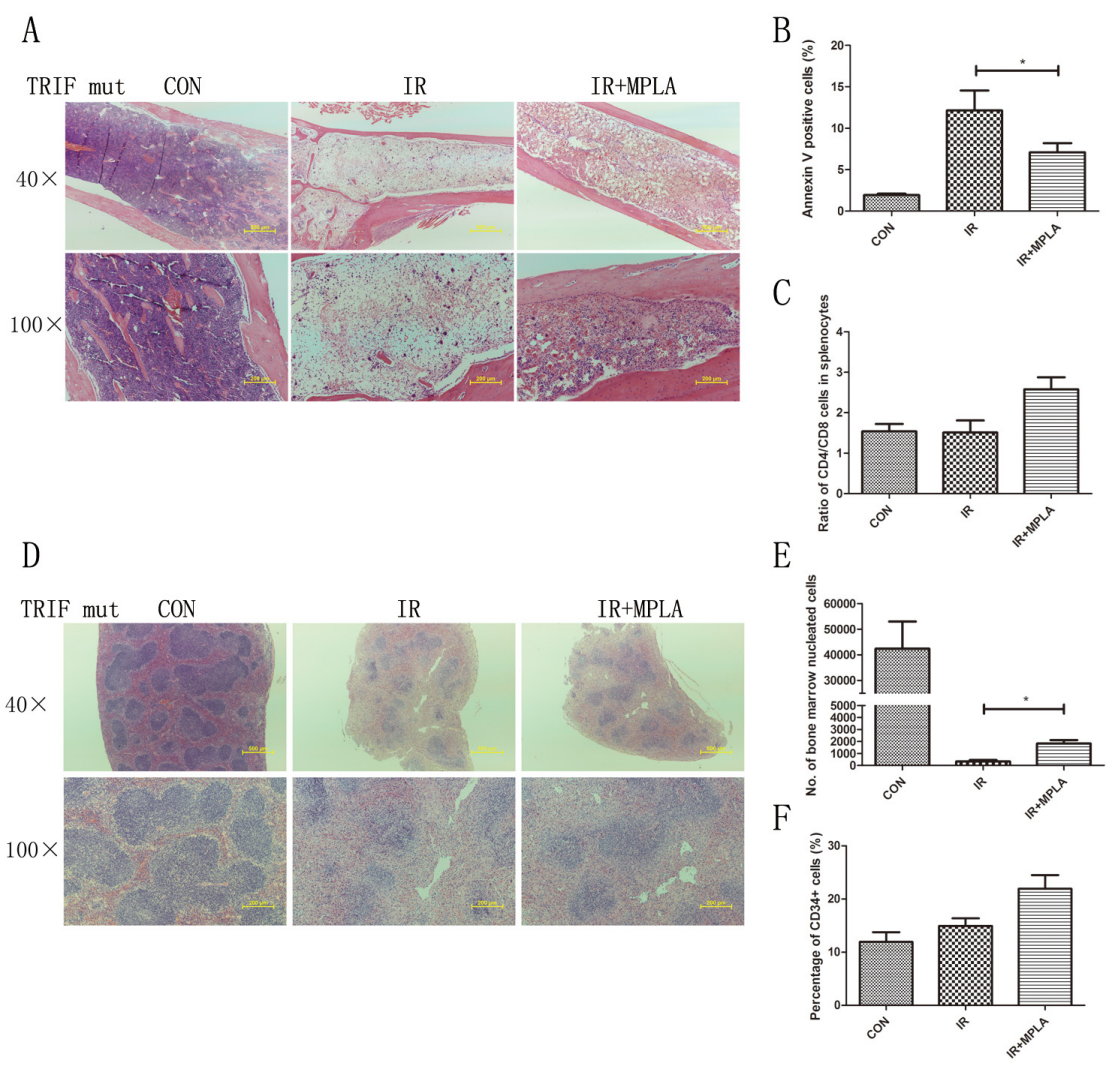
MPLA showed partial protective effects in TRIF mutant mice On the third day after 7Gy total-body irradiation, femurs **(A)** and spleen **(D)** were isolated from TRIF mutant mice and subjected to HE staining. At 24h after irradiation, cell apoptosis in splenocytes assay was analyzed **(B)**. Ratio of CD4+ and CD8+ splenocytes **(C)**, bone marrow nucleated cells **(E)** and percentages of CD34+ HSC **(F)** was analyzed by flow cytometry. *P<0.05, Vs IR groups. (n=10)

MPLA was reported as a potent TLR4 agonist, and we investigated the effects of MPLA in TLR4 knockout mice. We found that MPLA showed little protective effects against radiation in bone marrow and spleen (Figure 7A, 7D). We also checked the apoptosis of splenocytes, the number of bone marrow nucleated cells and CD34^+^ cells, and no significant difference was observed between single radiation group and MPLA pretreated group (Figure 7B, 7C, 7E). As previous studies showed that MPLA mainly triggers TRIF-biased signaling pathway, we investigated the effects of MPLA in TRIF mutant mice. We found that MPLA alleviated the structural damage of spleen and bone marrow (Figure 8A, 8D). In flow data, MPLA inhibited bone marrow nucleated cells loss and apoptosis in splenocytes, while showed little protection on CD34^+^ cells (Figure 8B, 8C, 8E).

### Radioprotective effects of MPLA on cultured cells was more dependent on MyD88 than TRIF

**Figure 9:**
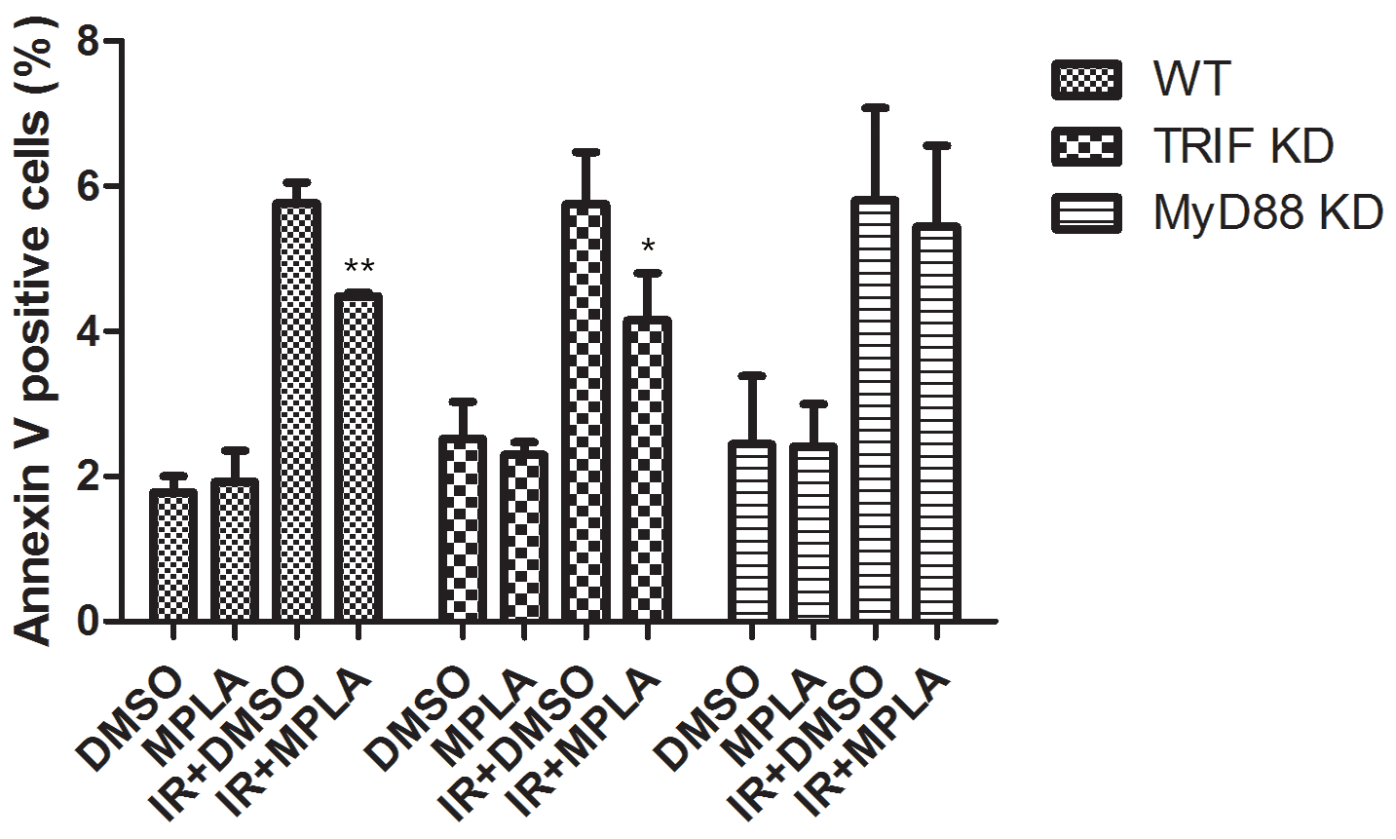
Radioprotective effects of MPLA were more dependent on MyD88 than TRIF At 48h after TRIF siRNA and MyD88 siRNA transfection, cells were treated with MPLA for 12h and subjected to irradiation. Cell apoptosis was detected by Flow cytometry analysis of Annexin V positive cells percentages at 24h after 8Gy irradiation. *P<0.05, Vs IR groups. (n=6).

Besides TRIF adaptor, MyD88 and its downstream signaling pathway also mediates the signaling of TLR4. To figure out the potential contribution of TRIF and MyD88, we used siRNAs to knock down TRIF and MyD88 in wild type RAW264.7 cells. We found that MPLA significantly inhibited apoptosis in TRIF knock down cells (Figure 9). While in MyD88 knock down cells, MPLA had no effect on cell apoptosis induced by irradiation (Figure 9). These data indicates that MyD88 signaling pathway mainly accounts for the radioprotective effects of MPLA.

## DISCUSSION

Toll like receptors (TLRs) have been shown to account for basal resistance to ionizing radiation. We have found that TLR4 was quite critical in radiation responses, and TLR4 ligand LPS effectively protected mice against ionizing radiation. However, for many tissues, LPS was too toxic to be used as a clinical radioprotector. In this study, we demonstrated that a low toxic TLR4 agonist, MPLA, effectively increased cell survival and inhibited apoptosis after irradiation. MPLA alleviated radiation injury on spleen, bone marrow, intestine and testis, which was dependent on TLR4 signaling pathway. MyD88 also accounts for the mechanism underlying radioprotective effects of MPLA.

To our knowledge, this is the first study reporting the radioprotective effects of MPLA, a potent TLR4 agonist currently used in clinic as a vaccine adjuvant. Our data showed that MPLA increased cell survival and inhibited apoptosis after exposure to irradiation. We observed that MPLA reduced the percentage of cells accumulated in G2/M phase. As radiation-induced cell apoptosis and cell cycle checkpoint activation are mainly attributed to response to DNA damage, we examined DNA damage repair in cells treated with MPLA with/without IR. Surprisingly, we found that MPLA promoted DNA damage repair by using a comet assay. And MPLA mainly activates TLR4 and NF-kB signaling pathway, which was also shown to be related to DNA damage repair. Then we checked the influence of MPLA on TLR4 and its downstream signaling pathway.

Exposure to IR induces severe damage to radiosensitive tissues including spleen, intestine, bone marrow and testis *etc.*. Our previous study proved that severe structural damage in bone marrow was presented in TLR4 (-/-) mice. In present study, we found that MPLA inhibited radiation injury on bone marrow, spleen, intestine and testis after exposure to IR. MPLA also increased the number of bone marrow nucleated cells, and inhibited cell apoptosis in splenocytes. Radiation induced an imbalance of CD4+ and CD8+ cells, which was also significantly alleviated by MPLA treatment. Next, we checked serum cytokines in different groups following irradiation. We found that MPLA plays anti-inflammatory roles in suppressing the level of IL-6 and TNF-α. MPLA also reversed radiation-induced Th1/Th2 shift in terms of IL-2 and IL-13. These data suggests that MPLA protects multiple tissues against IR, and exhibits immunomodulatory effects against IR.

As a low toxic derivative of LPS, MPLA has been proved as a potent TLR4 agonist by several studies. The effects of most TLRs are mediated by two main adaptors, TRIF and MyD88. We used TLR4 knockout mice and found the protective effects of MPLA were abrogated, indicating that radioprotective effects of MPLA are TLR4 dependent. Mata *et al.* reported that MPLA is a TRIF biased TLR4 agonist and induces gene expression mainly in the downstream of TRIF [15]. However, our experiments showed that the radioprotective effects of MPLA were not completely abrogated in TRIF mutant mice, suggesting alternative mechanism besides TRIF. A study on vaccine revealed that lack of either TLR4 or MyD88 had a significant impact on immune responses in mice immunized with MPLA-formulated vaccine [11], which provides the possibility that MyD88 also contributed to the signaling of MPLA. By using siRNA to knock down TRIF and MyD88 in cultured cells, we found that the protective effects of MPLA on cells depends more on MyD88 than TRIF.

Currently, several TLRs ligands have been proved to exert radioprotective effects, for example, CBLB502 for TLR5, CpG-ODN for TLR9, LPS for TLR4 and CBLB613 for TLR2/6 etc. Our group also demonstrated that Heat-Killed Salmonella typhimurium and Heat-Killed Mycobacterium tuberculosis protected mice against radiation through TLR signaling pathways [16, 17]. These findings shed new lights for application of TLR ligand in clinics. But efficacy and safety are equally important for an ideal radioprotector. As a derivative of LPS, MPLA retains the TLR4 recognizing structure, but reduced toxicity. And MPLA is currently approved for use as an adjuvant in Ceravix™ and Fendrix™ vaccine. It has been proved that MPLA is 10,000 less toxic than LPS [13], which results in a greater potential in clinical use. And over 300,000 human tests in vaccine have proved the safety of MPLA [14]. As a low toxic potent TLR4 agonist, MPLA shows great potentials in clinical uses for acute radiation exposure as well as the prevention of side effects resulting from radiotherapy.

In conclusion, our data showed that TLR4 agonist MPLA effectively protected cultured cells and radiosensitive tissues against irradiation through activating TLR4 signaling pathway. MPLA also inhibited inflammatory cytokines triggered by radiation. The radioprotective effects of MPLA were mainly dependent on TLR4-MyD88 signaling pathway. Our finding provides a novel safe and effective potential radioprotector for clinical application.

## MATERIALS AND METHODS

### Mice and treatments

All the experiments were approved by the Second Military Medical University, China in accordance with the Guide for Care and Use of Laboratory Animals published by the US NIH (publication No. 96-01). All experimental protocols were approved by the Experimental Committee of Second Military Medical University of China. Wild type C57BL/6 (6-8 weeks old, obtained from China Academy of Science, Shanghai, China), TLR2 deficient, TLR4 deficient and TRIF mutant mice from the same background (abbreviated to TLR2(-/-), TLR4(-/-), TRIF mutant respectively, obtained from Model Animal Research Center of Nanjing University) were used for the animal experiment. Maintained at 25±1 °C with food and water provided for free access, mice were housed in daily-changed individual cages. Either MPLA (1μg/mouse in 0.1ml physiological saline) or physiological saline (0.1ml /mouse) was delivered to the corresponding groups by intragastric administration 12h before exposure to total-body γ-radiation. At different time points after radiation exposure (1 day, 3 days, 7 days), mice were sacrificed for the following experiments to determine the radioprotective effects of MPLA. For animal survival experiments, mice (either physiological saline- or MPLA-pretreated) were observed and recorded every morning and evening for 30 days after radiation exposure.

### Cells and treatments

Human umbilical vein endothelial cells (HUVEC), Human embryonic hepatocytes (L02 cells) and murine macrophage RAW264.7 were purchased from American Type Culture Collection (Manassas, VA, USA), while the corresponding growth medium (DMEM for HUVEC and RPMI 1640 for L02) which were supplemented with both 10% fetal bovine serum and 1% penicillin-streptomycin solution (Hyclone, UT, USA) were obtained from PAA Laboratories. All cells were maintained at 37°C in a 5% CO_2_ humidified incubator. After MPLA treatment for 12h, cells were exposed to 8Gy γ-irradiation and used in the following experiments.

### Irradiation

The ^60^Co γ-rays in Radiation Center (Faculty of Naval Medicine, Second Military Medical University, Shanghai, China) was used for the irradiation exposure, which was described in previous studies [18, 19]. Mice were delivered a dose of 7Gy or 9Gy total-body γ-radiation at a rate of 1Gy/min for survival assay. For sub-total irradiation, mice were irradiated for 15Gy with their limbs and head shielded as previously described [20]. For tissue isolation, mice were exposed to 6Gy irradiation at the same dose rate.

### Cell viability assay (CCK-8 assay)

A Cell Counting Kit-8 (Beyotime, Shanghai, China) was utilized to detect cell viability according to the manufacture’s protocol. L02 and HUVEC cells were seeded evenly in 96-well plates in triplicate and after adherence (at least 8h later) they were treated with either MPLA (1μg/ml) or PBS. After 12h pretreatment, cells in all the groups were exposed to 8Gy irradiation. And then cell viability assay was performed at 48h post-irradiation.

### Apoptosis assay and cell cycle analysis

Twenty four hours after irradiation, 2×10E5 cells in 50μl of binding buffer were incubated with 5μl Annexin V-fluorescein isothiocyanate (Annexin V-FITC) for 20min using an Apoptosis Detection Kit (Invitrogen, Carlsbad, California, USA) according to the manufacturer’s instruction. Before flow cytometry sampling, 2μl Propidium Iodide (PI) was added immediately. For cell cycle analysis, cells were collected at 12h post-irradiation and were tested by using a PI staining assay described previously [21]. To determine the contribution of MyD88 and TRIF to radioprotection by MPLA, we used siRNAs to knock down these two molecules in RAW264.7 cells. The sequences of siRNA were listed below: TRIF, sense: GCUAUGUAACACACCGCUGTT; antisense: CAGCGGUGUGUUACAUAGCTT. MyD88, sense: CCUGCAAAGUAAGGAAUGUTT; antisense: ACAUUCCUUACUUUGCAGGTT. At 72h after siRNA transfection, cells were irradiated and 24h later, cell apoptosis were measured.

### Comet assay

DNA double-strand breaks were determined by the neutral comet assay previously described with modification [22]. Firstly, we prepared the slides by immerse the dust-free slides into molten 1% normal melting agarose (NMA) and wiping one side clean immediately. All of the slides were pre-coated ahead of time to ensure they turn to be thoroughly dry for next use (one day ahead). Secondly, the concentration of the prepared single-cell suspension in the ice cold Ca^2+^-free and Mg^2+^-free PBS was adjusted to 2×104 cell/ml, then 0.4 ml of which was dipped into the 1.2 ml LMA which was kept at 40°C within the water bath. Thirdly, 1.2 ml of the cell suspension was mixed and rapidly pipetted onto the surface of a pre-coated slide. Fourthly, once solidified the gels began to be lysed in the neutral lysis solution (58.44g NaCl, 5.584g Na_2_EDTA and 0.61g Tris into 500ml double distilled water, PH 8.2-8.5, Triton X-100 was added to the terminal concentration of 1% before use) in the dark overnight. After that, slide were submerged gently for 30 min in the TBE rinsing buffer (0.744g Na_2_EDTA, 10.902g Tris and 5.564g boric acid into 500ml double distilled water, PH 8.2-8.5) for 3 times and then electrophoresed at 4°C in the dark for 25 min at 25 V and 7 mA in fresh TBE. Finally, the ddH2O (double distilled water)-washed gels were dyed with PI (10 μg/ml) for 20 min and subsequently rinsed gently with ddH2O. Finally, all the gels were examined via a fluorescent microscope (Olympus BX60) under a 10× objective lens. At least 100 comet images from each slide were analyzed for several features containing DNA Content, Tail Length, Olive Tailmoment and Tailmoment using special analysis software named CASP 1.2.3b2 (CASPlab, Wroclaw, Poland).

### Western blot analysis

Proteins were obtained from cell lines at 0.5h, 8h post-irradiation using ProtecJETTM Mammalian Cell Lysis Reagent (Fermentas, Vilnius, Baltic, Lithuania) according to the manufacturer’s instruction. Then the samples were analyzed by western blotting with chemiluminescent detection as described previously [23]. We detected MyD88 (Abcam, US; 1:1000), TRIF (Proteintech, Wuhan, China; 1:1000), p-p65 (Cell signaling tech, USA: 1:1000), p-IKKβ (Cell signaling tech, USA: 1:1000), p-IkBα (Cell signaling tech, USA: 1:1000), p-Jnk, p-Erk, p38 and GAPDH (sampler kit from Cell signaling technology, US; 1:1000) using secondary antibodies (1:1000) purchased from Cell Signaling Technology.

### Enzyme-linked immunosorbent assay (ELISA) assay

Serum was isolated from blood drawn from angular vein venous just before the animal was sacrificed (3rd days after radiation) and subjected to ELISA assay. The concentration of cytokines including IL-1β, IL-2, IL-4, IL-6, IL-10, IL-12, TNFα and IFNγ were determined following the manufacturer’s instructions. (Westang Tech., Shanghai, China) [24].

### Flow cytometric analysis of splenocytes and bone marrow cells

On the 3rd day after irradiation, single-cell suspension of bone marrow and spleen were analyzed using flow cytometry as described previously [25]. Immediately after the blood withdrawing, mice were sacrificed by dislocation of cervical vertebra. Femurs, spleens and testis were removed from each mouse. For bone marrow cells (BMC) count, the primary single cell suspensions of one femur for each mouse were filtered through a 200 mesh sieve, within which the red blood cells were lysed and eliminated thoroughly. In subsequence the whole number of nucleated cells were counted totally via flow cytometry. Hemopoietic stem cells were also analyzed with flow cytometry through staining with CD34-FITC and B220-PE antibodies. In addition, isolated splenocytes were incubated with CD4^+^- and CD8^+^- antibodies and subjected to the flow cytometric analysis as well.

### Histopathological studies

Spleens, intestine, testis and femurs (one for each subject) were isolated and fixed with 4% paraformaldehyde on 1st, 3rd and 7th day post-irradiation. Subsequently, the samples were embedded in paraffin, cut into thin sections (4μm thick) and stained with the hematoxylin-eosin (H&E) for the final histopathological discriminations performed in a double blind method [26-28].

### Immunofluorescence staining of p65 and nuclear factor-kappaB (NF-kB) luciferase assay

The translocation of the NF-kB p65 protein was assessed by an immunofluorescence assay as described previously with a little modification [29]. The L02 and HUVEC cells, seeded on coverslips glasses in 6-well plates (2×10^5^/well) for 24h, were treated with MPLA and radiation. At 0, 0.5, 2h post-irradiation, cells were fixed with 4% paraformaldehyde for 20 min at room temperature and then washed with PBS. After incubated with 0.5% Triton-X100 (Sigma) and 5% goat serum, cells were incubated with NF-kB p65 primary antibody (Santa Cruz, US; 1:200, 2h) and secondary antibody (1:1000, 1.5h). At last, the nuclei were counterstained with 4’,6-diamidino-2-phenylindole (DAPI) for 10 min (1:2000). Fluorescence images were captured by a fluorescent microscope (Olympus America Inc., Center Valley, PA, USA) connected to a Retiga 2000 R digital camera (QImaging Inc., Surrey, BC, Canada). At least 100 cellular images were examined each group by means of ImageJ software (version 1.44p, Wayne Rasband, National Institute of 113 Health, USA). NF-kB activity was determined by using NF-kB luciferase reporter assay (Genemeditech, Shanghai, China) according to manufacturer’s instruction.

### Statistical analysis

Data were expressed as mean±standard deviation (S. D.) unless indicated otherwise. Generally, a two-tailed unpaired Student’s t-test was used to determine the statistical significance between groups. The comparison of the survival curves was analyzed by Kaplan–Meier analysis followed by the Mantel-Cox test. A P-value less than 0.05 was considered to be significant. Statistical Product and Service Solutions (SPSS) Software, version 21 (SPSS Inc., Chicago, IL, USA) was utilized to all statistical analyses. GraphPad Prism 5 Software (GraphPad software Inc, California, USA) was used to conduct for graphs.

## Abbreviations

MPLA: Monophosphoryl lipid A
TLR: Toll like receptor
IR: Ionizing radiation
LPS: lipopolysaccharide
DSB: double-strand breaks
TRIF: Toll/IL-1R domain-containing adaptor inducing IFN-β
MyD88: myeloid differentiation factor 88 (MyD88)
